# Barrier Properties and Hydrophobicity of Biodegradable Poly(lactic acid) Composites Reinforced with Recycled Chinese Spirits Distiller’s Grains

**DOI:** 10.3390/polym13172861

**Published:** 2021-08-26

**Authors:** Zhi-Jun Chen, Chi-Hui Tsou, Meng-Lin Tsai, Jipeng Guo, Manuel Reyes De Guzman, Tao Yang, Chen Gao, Yan Lei, Pei-Wen Gan, Shuang Chen, Lian-Jie Tu, Chang-Lei Qu, Ruo-Yao Wang, Chin-San Wu

**Affiliations:** 1 Material Corrosion and Protection Key Laboratory of Sichuan Province, School of Materials Science and Engineering, Sichuan University of Science and Engineering, Zigong 643000, China; czj2356068001@163.com (Z.-J.C.); Bb7211260@163.com (J.G.); manuelrdg@yahoo.com (M.R.D.G.); y19130115495@126.com (T.Y.); s19871016gc@sohu.com (C.G.); yanlei224@suse.edu.cn (Y.L.); peiwen@suse.edu.cn (P.-W.G.); cs68811190@163.com (S.C.); tfzhhy@163.com (L.-J.T.); quchangleixuexi@shse.edu.cn (C.-L.Q.); 2 Sichuan Yibin Plastic Packaging Materials Co. Ltd., Yibin 644007, China; 3 Sichuan Golden-Elephant Sincerity Chemical Co. Ltd., Meishan 620010, China; 4 Sichuan Zhixiangyi Technology Co. Ltd., Chengdu 610051, China; 5 Department of Materials Science and Engineering, National Taiwan University of Science and Technology, Taipei 10607, Taiwan; mltsai@gapp.ntust.edu.tw (M.-L.T.); steven5202001@yahoo.com.tw (R.-Y.W.); 6 Department of Applied Cosmetology, Kao Yuan University, Kaohsiung 82101, Taiwan

**Keywords:** poly(lactic acid), Chinese spirits distiller’s grains, barrier, mechanical behavior, hydrophobicity, biodegradable properties

## Abstract

Adding natural biomass to poly(lactic acid) (PLA) as a reinforcing filler is a way to change the properties of PLA. This paper is about preparing PLA/biomass composites by physically melting and blending Chinese Spirits distiller’s grains (CSDG) biomass and PLA to optimize the composite performance. Composites of modified PLA (MPLA) with varying amounts of CSDG were also prepared by the melt-mixing method, and unmodified PLA/CSDG composites were used as a control group for comparative analysis. The functional groups of MPLA enhanced the compatibility between the polymer substrate and CSDG. The composite water vapor/oxygen barrier and mechanical properties were studied. It was found that the barrier and mechanical properties of MPLA/CSDG composites were significantly improved. SEM was adopted to examine the tensile section structure of the composites, and the compatibility between the filler and the matrix was analyzed. An appropriate amount of CSDG had a better dispersibility in the matrix, and it further improved the interfacial bonding force, which in turn improved the composite mechanical properties. X-ray diffraction, thermogravimetric analysis, and differential scanning calorimetry were conducted to determine the crystalline properties and to analyze the stability of the composites. It was found that the CSDG content had a significant effect on the crystallinity. Barrier and biodegradation mechanisms were also discussed.

## 1. Introduction

Research and development on environment-friendly polymers and polymer composites based on renewable resources has attracted increasing attention [1,2]. Natural [3,4] and various functional materials used in manufacturing have gradually replaced traditional ones [5,6] because of their clean, easy-to-use, harmless, and environment-friendly features, and their usage can reduce energy consumption [7] and environmental pollution [8,9]. Poly(lactic acid) (PLA) can be naturally metabolized in the human body into carbon dioxide and water. It is called “an environmental recycling material of the 21st century” [10,11,12]. PLA/natural plant fiber composites have considerable performance and excellent biodegradability; they are known as “green” composites and are currently a hot spot in the field of biodegradable materials.

Cellulose sources are rich and diverse, including crop straw, bagasse, wood, bamboo, and corn cobs, and other agricultural waste [13,14]. Filling PLA with natural biomass fibers to prepare composites reduces the density of the polymer matrix, improves the strength and biodegradability of the composites, and greatly reduces the cost. Application fields include construction and civil engineering, sports and entertainment products, medical equipment, bionic products, and home office supplies, and they have expanded to various fields such as aerospace, national defense, and military industry [15,16]. This expansion not only improves the added value of plant fibers but also makes full use of degradable PLA to replace original nonrenewable petroleum-based plastics. The use of degradable plastics is of great significance to the environment and human development [17].

Zhao et al. [18] carried out surface modification treatment on rice straw fiber (RSF) and on combined modified and unmodified RSF with PLA, and they compared and analyzed different samples. The results showed that modified RSF had obvious improvement in the composite tensile strength. When the RSF content was 30%, the elongation of the modified sample was 60.8% higher than that of the unmodified one. Yaacab et al. [19] used rice straw powder (RSP) as filler and PLA as matrix, and they mixed them with hot pressing, followed by high-pressure cooling, to obtain PLA/RSP biocomposites. The results indicated that the tensile strength of PLA/RSP composites decreased as the RSP content increased, but the tensile modulus and stiffness increased significantly. Increase in RSP reduced the thermal stability and crystallinity.

Way et al. [20] applied some maple wood fiber (WF) as a reinforcing filler to prepare PLA/WF composites by melt blending. They studied the composite mechanical and thermodynamic properties, and they reported that the interfacial compatibility between PLA and WF was enhanced, and the mechanical properties of the composites were improved as a result of the chemical treatment with a silane coupling agent. Yusoff et al. [21] adopted an effective method to extract bamboo fiber and subsequently characterized its mechanical properties. Bamboo, coconut, or kenaf fiber was used as reinforcement to prepare an environment-friendly composite with PLA. The mechanical properties were tested, and the fiber structure was observed using SEM. The results showed that when three kinds of fibers were added, the mechanical properties or strength of the composites were improved. Wassamon et al. [22] combined natural fibers, such as bamboo, vanilla, and coconut, with modified PLA to prepare a new type of environment-friendly functional materials. Their study demonstrated that the rigidity of composite materials increased significantly.

Bourmaud et al. [23] used maleic anhydride-grafted PLA (PLA-g-MAH) as a compatibilizer in PLA/reed fiber composites, and they found that adding the compatibilizer enhanced the reed fiber adhesion to the polymer matrix. The degree of fiber dispersion significantly improved the compatibility between the fiber and PLA. Zhang et al. [24] chose PLA-g-MAH as a compatibilizer for wood flour-reinforced PLA composites, and they observed that when the mass fraction of PLA-g-MAH was 30 wt %, the mechanical properties were significantly improved compared with those of the composites without a compatibilizer.

A category of biomass is a by-product mainly obtained from wine industries, known as distiller’s grains, one type of which is referred to as Chinese Spirits distiller’s grains (CSDG). The majority of CSDG is burned or buried as waste, which not only wastes resources but also causes environmental pollution. However, biomass can be used as fillers to make biodegradable materials. For example, plant fibers can act as nucleating agents in the crystallization process of different thermoplastic polymers and interfere with their supramolecular structure [25]. However, plant fibers also have disadvantages such as poor water resistance and poor plasticity. CSDG can be an alternative, as it is also an important part of biomass, and it is likely to replace glass fibers, inorganic fibers, and other fillers commonly used in plastics to prepare environment-friendly composites. Therefore, CSDG may be combined with PLA to improve the water resistance of composites. At the same time, it may expand further the application of green plastics or the application fields of natural fibers.

CSDG, an unfermented grain, is a by-product from ethanol fuel industries. At present, there is little research on the application of CSDG in PLA composites. Only Lu et al. [26] prepared biodegradable composites in 2014 by mixing PLA with dried distiller’s grains. Only the two materials—PLA and dried distiller’s grains—were mixed. Neither PLA nor distiller’s grains was modified, and the change in mechanical strength was not mentioned in the study. It was only estimated that the mechanical properties should not be ideal. Moreover, the research was mainly focused on biodegradable and thermal properties, and the content of distiller’s grains was only 20%. There is still much room for discussion and further studies. Interestingly, CSDG contains a lot of grains, and it is more complicated than distiller’s grains in general. In addition, CSDG has never been reported in studies dealing with PLA. In this present work, CSDG was used as a reinforcing filler, and a composite material was obtained by melt blending it with PLA. A modified form of PLA (MPLA) was also considered to improve the interfacial compatibility between CSDG and PLA. The hydrophobicity, biodegradability, as well as the mechanical, barrier, crystallization, and thermodynamic properties of the composites were characterized and analyzed. The effect of varying amounts of CSDG on the physical and mechanical properties of the composites was investigated to explore the potential application of CSDG in the field of green plastics.

## 2. Experimental

### 2.1. Experimental Materials

A 2002D grade of PLA was used in this present study. It was from Natureworks LLC (Minnesota, MN, USA), with a number molecular weight of 100,000 g/mol. The modification method to form MPLA was similar to that in previous studies [27,28], and it involved grafting maleic anhydride to PLA (thus, maleic anhydride-grafted PLA was produced). The grafting rate of MPLA was about 1.2%, and the tensile strength of MPLA was 37.08 ± 0.7 MPa.

### 2.2. Preparation of Composites

PLA/CSDG and MPLA/CSDG composites were prepared, with the CSDG concentration varied from 10 to 50%. The proportion of components in each material is shown in Table 1. The steps of preparing the composite materials are as follows:

(1) Drying of materials: Before the sample preparation, PLA and CSDG were placed in a vacuum oven at a drying temperature of 85 °C for a drying time of 8 h.
(2) Preparation of PLA/CSDG or MPLA/CSDG composites: A torque rheometer (HAAKE PolyLab OS) was used. The conditions at which it was operated were at a temperature of 180 °C and a speed of 100 rpm. First, PLA or MPLA were added to melt them for 1 min. Then, CSDG powder was added, and its content was varied (10, 20, 30, 40, and 50 wt %). The two materials were blended for 10 min to prepare PLA/CSDG or MPLA/CSDG composites with different concentrations of CSDG.

The prepared composite was hot-pressed using a vulcanizer at a temperature of 180 °C for 10 min. The material was taken out of the vulcanizer to left to cool. A custom-made cutter was used to cut the material and form a dumbbell-shaped sample, which was used for testing and characterization.

### 2.3. Fourier Transform Infrared Spectroscopy

The infrared absorption spectrum of a material can be obtained by detecting infrared absorption, also known as molecular vibration. Before the test, samples were dried at 80 °C and were cut into pieces. The spectral analysis of the samples was characterized by Fourier transform infrared (FTIR) (NICOLET 6700, Thermo Scientific, Waltham, MA, USA) spectroscopy to obtain the characteristic peaks of the samples.

For the test parameter setting, the test wavelength range was 4000–500 cm^−1^.

### 2.4. Mechanical Properties

A microcomputer-controlled electronic universal testing machine (FBS-10KNW, Xiamen Forbes Testing Equipment Co. Ltd., Xiamen, China) in plastic-film tensile test mode was used to determine the mechanical properties of the PLA/CSDG and MPLA/CSDG composites. Tensile strength referred to the ratio of the maximum load P before the test sample broke to the cross-sectional area of the test sample under a specific test temperature and humidity. A tensile load was applied along the axial direction. Tensile strength is usually expressed as *δ_t_*, and its calculation formula is:

(1) $$\delta_{t}=P/(b\times d)$$

where *P* = maximum breaking load, N; *b* = sample width, mm; *d* = sample thickness, mm.

The elongation at break was the relative elongation of the test sample when it broke, and was calculated by the following formula:

(2) $$\varepsilon_{t}=(F-G)/G\times 100\%$$

where *G* = distance between marking lines of sample, mm; *F* = distance between marking lines when sample broke, mm.

The characterization parameter setting is as follows: sample in dumbbell shape; 1000 N sensor range; 2 mm/min set pulling speed.

### 2.5. X-ray Diffraction

X-ray diffraction (XRD) technology is the most important structural test method. The prepared sample was analyzed using XRD (D2 PHASER, Bruker, Germany), and corresponding peaks for PLA/CSDG and MPLA/CSDG composites were obtained.

The test parameter setting is given as follows: working voltage = 40 kV, working current = 30 mA, scanning area = 0.02°/s, and step length = 10–90°.

### 2.6. Morphology Characterization

Scanning electron microscopy (SEM) was used to observe the fracture surface morphology of tensile samples taken from composite materials. SEM is an excellent technique for obtaining morphological information by scanning the surface with electron beams. It can generate high-resolution 3D surface images to describe the surface structure of samples. The generated SEM images clearly depicted the morphology of PLA/CSDG and MPLA/CSDG composites and the distribution of CSDG in them. Before SEM tests were conducted, samples were attached to a support and sputtered with a coating or layer of gold. Then, SEM (VEGA3SBU, TESCAN, Brno, Czechia) was operated to characterize the cross-sectional morphology of PLA/CSDG and MPLA/CSDG composites.

The SEM test parameter setting is indicated as follows: time of spraying gold on samples, 30 s; working current, 5 mA; scanning or acceleration voltage, 3 kV.

### 2.7. Thermal Analysis

Differential scanning calorimetry (DSC) or a thermal analyzer (DSC200 F3, NETZSCH, Selb, Germany) operated under nitrogen gas was used to test the crystallization temperature and crystallinity of composite films. The sample to be tested was placed in a crucible, and a reference material was placed in another crucible. Then, the two crucibles were heated at the same rate. The sample had to undergo melting, crystallization, oxidation, and degradation processes, so as to obtain the following parameters: T crystallization, T oxidation, T melting, and T decomposition.

(3) $$X_{C}=(\frac{\Delta H}{\Delta H_{0}\times wt\%})\times 100\%$$

In Equation (3), *X_C_* = degree of crystallinity, Δ*H* = test sample heat of fusion, Δ*H*_0_ = heat of fusion of a pure PLA substrate when the crystallinity is 100%, and *wt*% = percentage of PLA in the sample. PLA with 100% crystallinity has a theoretical enthalpy of 93.7 J/g.

The test parameter setting is given as follows: heating rate of 25 °C/min (heating was from room temperature to 200 °C); maintaining 200 °C constant for 10 min; reduction of temperature to 2 °C; finally, increase of temperature to 200 °C at a rate of 10 °C/min. The sample crystallization temperature was measured.

### 2.8. Thermogravimetric Analysis

Thermogravimetric analysis (TGA) is a method used to study the composition and thermal stability of materials. Results were obtained by measuring changes in the sample weight. After data were obtained, DTG (differential TG) could be analyzed to determine the degree of change in the sample weight as the temperature increased (i.e., mass loss rate). A thermal analyzer (STA 409PC, Netzsch Company, Erlangen, Germany) was used, wherein both the test samples and the balance were under nitrogen flow. Subsequently, TGA curves were obtained.

The test parameter setting is given as follows: nitrogen was used as shielding gas with a flow rate of 70 mL/min; starting from room temperature, the temperature was increased at a rate of 10 °C /min, and the heating was stopped when it reached 600 °C. Origin software was used to make a diagram for temperature–mass loss ratio and for temperature–DTG data. At the same time, the following were listed: initial degradation temperature, maximum temperature, and total weight loss rate.

### 2.9. Oxygen Barrier Properties

The penetration of small gas molecules through defect-free films is a molecular diffusion process. First, small gas molecules would be adsorbed and dissolved on the film. Under the action of concentration gradient, the gas molecules would then diffuse from a high concentration to a low concentration, when the gas concentration increases to a certain level. Finally, they would diffuse out on the other side of the film. A differential pressure gas permeation meter (VAC-V2, Labthink Instrument Co. Ltd., Jinan, China) in proportional mode was used to test PLA/CSDG and MPLA/CSDG composites in terms of their oxygen barrier performance, which is expressed as oxygen permeability. Its unit was cm^3^/m^2^ d Pa.

The test parameter setting was as follows: temperature of test chamber was set to 25 °C, film thickness = 0.08 mm, test environment contained oxygen + nitrogen, judgment ratio = 10%, and relative humidity was 100%.

### 2.10. Water Vapor Barrier Properties

A water vapor transmission (WVT) rate test system (W3/060, Labthink Instrument Co. Ltd.; Jinan, China) in standard mode was used to test the water vapor barrier properties of PLA/CSDG and MPLA/CSDG composites. The WVT rate was calculated using the equipment software. The WVT performance includes two meanings: water vapor permeability (WVP) and WVT coefficient. These two meanings are different, but they can both be used to indicate the ability of water vapor to pass through a certain material. The WVT rate indicates the weight of water vapor passing through the material in a certain period of time, under certain temperature and humidity conditions. The WVT coefficient is the standard value of WVT calculated by the system. It is used for comparing different test results and standard values of different samples. The result was expressed as WVP in g/m^2^/d.

The test parameter setting was as follows: temperature of test chamber, 25 °C; film thickness, 0.08 mm; test interval, 30 min; humidity, 50%.

### 2.11. Contact Angle Test

A contact angle measuring instrument (JC2000D, Shanghai Zhongchen Digital Technology Equipment Co. Ltd., Shanghai, China) was used to test and characterize PLA/CSDG and MPLA/CSDG composites in terms of their hydrophilic properties. Contact angle is a measure of the degree of wetting a solid with a liquid. If the test value is less than 90°, the surface of the sample is hydrophilic, and the smaller the value, the better the wettability of the liquid to the sample. If the test value is greater than 90°, the sample surface is hydrophobic, and the larger the value, it means that it is not easy for the liquid to wet the sample.

A microsyringe was used to extract 2 μL of distilled water, which was dropped on a sample surface. Contact angles were recorded following a five-point fitting method. For all samples, three measurements were taken, and the average was calculated.

The test parameter setting was as follows: sample thickness, 0.08 mm; test time was 0, 3, and 5 s; repetition for each test, 3 times.

### 2.12. Water Absorption

Water uptake (WU) was measured according to standard methods [29]. Samples were cut into 2 cm × 2 cm and dried to constant weight in an oven at 105 °C. Before tests were done, samples were weighed and soaked in distilled water for 24 h at room temperature. Then, they were taken out of the water, rid of adhering water drops, and weighed. Water absorption or *WU (g/g)* was calculated using Equation (4). The sample weight before soaking was represented as *m*_0_ (g), and *m_f_* was the sample weight after soaking (g).

(4) $$WU(g/g)=\frac{m_{f}-m_{0}}{m_{0}}=100\%$$

### 2.13. Degradation Performance Test

The dimension of samples was 2 cm × 2 cm. They were dried in an oven at 105 °C until constant weights were recorded. Before the degradation test was conducted, samples were weighed and buried in soil at room temperature. The test cycle was 180 days. Samples were retrieved from the soil every 30 days, and they were washed, dried, and weighed. The degradation rate was calculated according to Equation (5). *C*_0_ was the initial sample weight (g), and *C_t_* was the sample weight after the degradation (g).

(5) $$\mathrm{degradation}\;\mathrm{rate}\;(\%)=\frac{C_{0}-C_{t}}{C_{0}}\times 100\%$$

## 3. Results and Discussion

### 3.1. Fourier Transform Infrared Spectra

**Figure 1:**
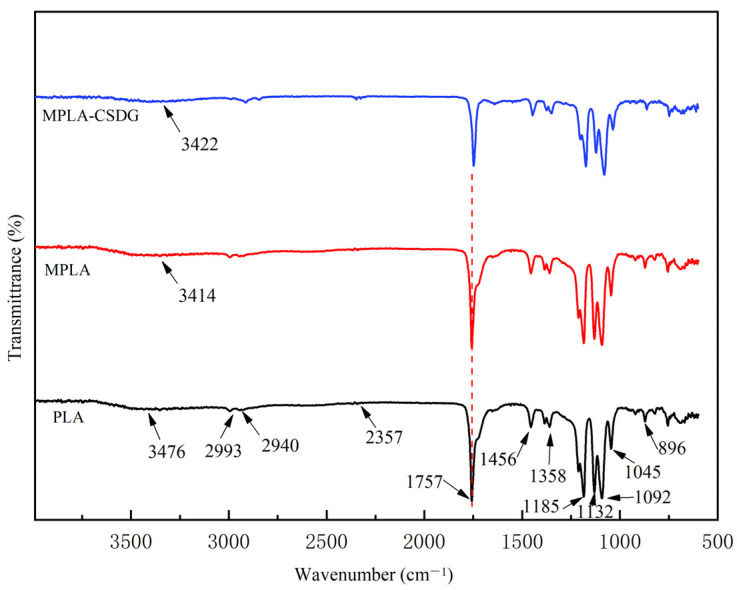
Fourier transform infrared spectra of PLA, MPLA, and MPLA/CSDG.

Figure 1 represents the infrared spectra of PLA, MPLA, and MPLA/CSDG composites. The infrared characterization of these three materials at around 2993 and 2940 cm^−1^ wavenumbers gave a description of symmetric and asymmetric vibration peaks of −CH− in the PLA structure. Pure PLA and the composite material had an obvious absorption peak at about 2357 cm^−1^, corresponding to the characteristic absorption peak of carbonyl CO_2_, which may be due to the inevitable air exposure during the process of placing the sample into the detector. The wavenumber at 1430 cm^−1^ corresponded to –CH– scissor bending vibration, and that at 1370 cm^−1^ referred to the characteristic absorption peak of –CH_3_–. Absorption peaks at 1185, 1132, 1092, and 1045 cm^−1^ denoted −C−O− stretching vibration peaks. Due to the presence of hydroxyl at the other end of the PLA molecular structure, there would be a weak absorption peak at 3450 cm^−1^, which is not evident (Figure 1). The main reason is that the hydroxyl absorption peak intensity of PLA was relatively small. Figure 1 does not show it clearly, but when biomass fillers that contained a large number of hydroxyl groups were added to the MPLA matrix, the peak position of the hydroxyl groups slightly shifted to the right. This implies that the hydroxyl groups on the surface of the biomass fillers could interact with PLA. That is, the molecular segments of MPLA and CSDG may be bound together. On the other hand, the position of the characteristic carbonyl peak in PLA was about 1757 cm^−1^, and the characteristic peak of carbonyl in the MPLA composite shifted to the right. This also implies that the hydroxyl group on the surface of MPLA could interact with the carbonyl group in PLA. On the basis of the above analysis, along with the research by Wu and Tsou [30] on PLA and rice husk regarding a modification treatment through the use of a coupling agent, the PLA component indicated a behavior similar to the shift of carbonyl characteristic peaks. This interaction could improve the related properties of biomass filler and PLA composite materials.

### 3.2. Data on Mechanical Properties

**Figure 2:**
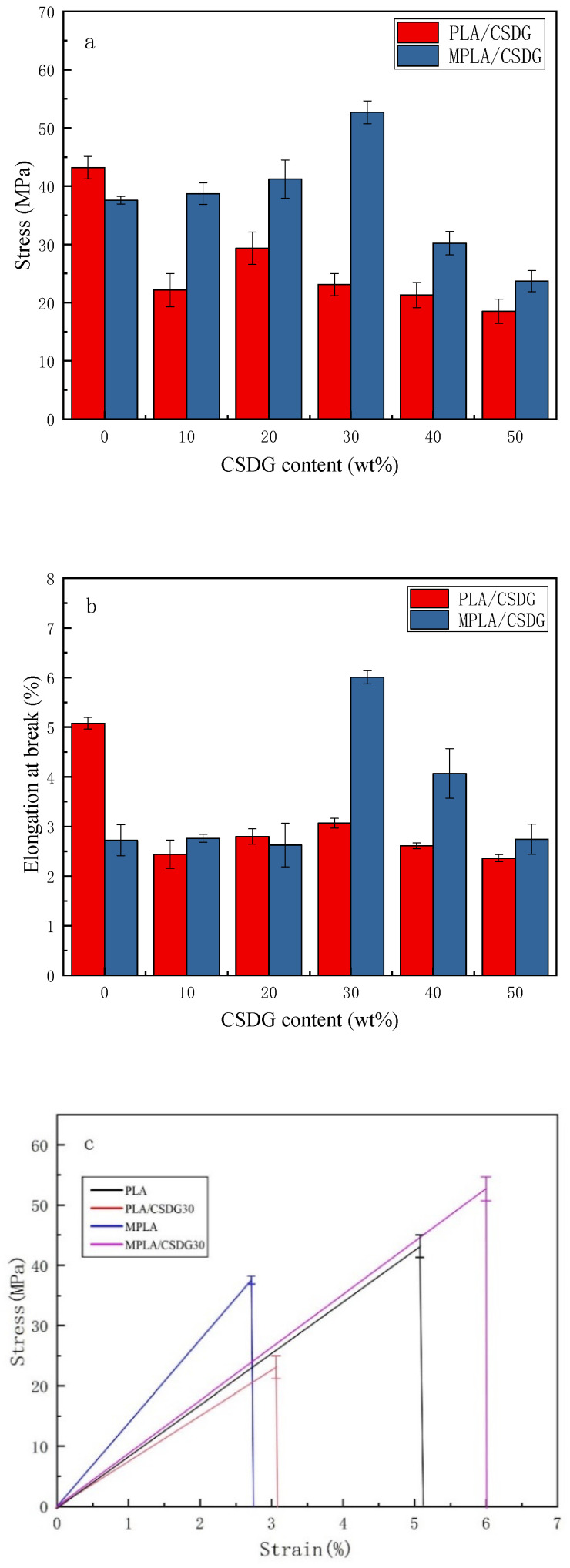
Mechanical properties of PLA/CSDG and MPLA/CSDG composites: (**a**) tensile strength; (**b**) elongation at break; (**c**) stress–strain curves.

Figure 2 and Table 2 provide test results on the influence of different amounts of CSDG on the mechanical properties of PLA/CSDG and MPLA/CSDG composites. Figure 2a depicts tensile strength as a function of the CSDG content, Figure 2b presents data on elongation at break, and Figure 2c plots stress–strain curves. The tensile strength of pure PLA was indicated to be 43.2 MPa. When the content of CSDG was 10%, the tensile strength of the composite material was reduced to 22.15 MPa. At 20% CSDG, the tensile strength increased to 29.34 MPa, which was the highest value. When the amount of added CSDG reached 50%, the tensile strength was significantly reduced to 18.5 MPa. Compared with the overall strength of pure PLA, that of the PLA/CSDG composites was lower. In the tensile strength data for MPLA/CSDG composites, the tensile strength of the MPLA composite with 10% CSDG was 38.73 MPa. When the CSDG content was increased to 20% and 30%, the tensile strength increased, and the maximum value of 52.68 MPa was reached at the 30% content. However, when 40% CSDG was added, the tensile strength was greatly reduced to 30.2 MPa. Stress–strain curves in Figure 2c indicated a significant difference between PLA and MPLA composites containing 30% CSDG—the tensile strength and elongation at break of the MPLA/CSDG composites were significantly higher than those of the PLA/CSDG composites.

When CSDG was added to PLA, the elongation at break showed a trend of an initial large decrease, which was followed by a slight increase. The elongation at break reached 3.07% for the sample with 30% CSDG, which was higher than that for the other samples but still lower than that of the pure PLA sample. For the MPLA/CSDG composites, it is apparent that the trend of elongation at break differed much from that for the PLA/CSDG composites. When the content was 30% CSDG, the elongation at break for MPLA/CSDG reached 6.01%, which is the highest among the samples, and it was 1.2 times higher than that for pure PLA.

According to the analysis, the tensile strength and elongation at break of the MPLA/CSDG samples were higher overall than those of PLA/CSDG. These data verify the results from the FTIR analysis, which indicated that the mechanical properties of composite materials became stronger due to the internal interaction. As illustrated by the SEM morphology, the compatibility between CSDG and the PLA matrix became poor when the addition of CSDG was too high, resulting in decreased toughness of the composite material and increased brittleness. In addition, when CSDG increased to a certain level, agglomeration appeared in the film, causing brittle fracture to be more likely to occur [31]. This explanation is consistent with the results obtained by Zhao et al. [32]. In other words, improving the interfacial adhesion is of great significance in enhancing the mechanical properties of composite materials.

### 3.3. X-ray Diffraction Patterns

**Figure 3:**
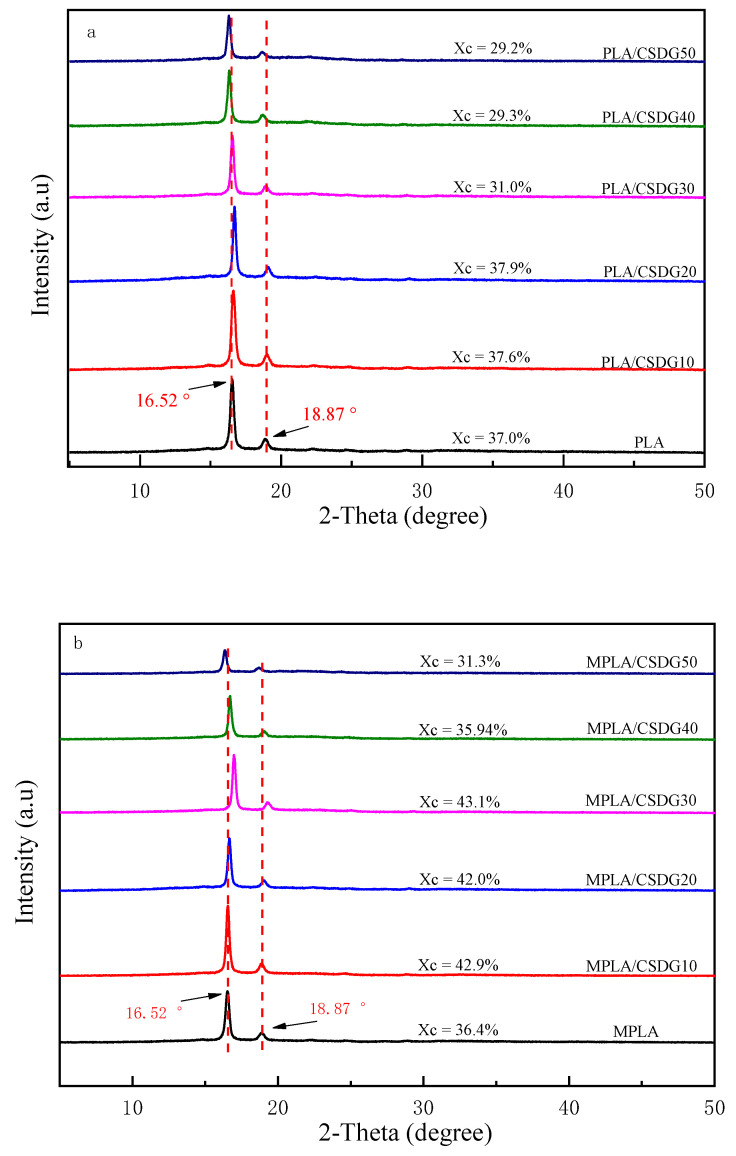
X-ray diffraction patterns: (**a**) PLA/CSDG; (**b**) MPLA/CSDG.

With the help of the XRD patterns shown in Figure 3, PLA, MPLA, and two sets of composite materials were analyzed. Figure 3a is the XRD spectra of PLA/CSDG composites, and Figure 3b is the XRD spectra of MPLA/CSDG composites. Different proportions of CSDG in the composites affected the crystallization and the degree of enhancement in the structure and other properties.

There are obvious sharp diffraction peaks in PLA at 2θ = 16.52 and 18.87°, which refer to (200/110) and (203) planes corresponding to typical α crystals of PLA [33,34]. Throughout the spectral curve, PLA/CSDG with 10% and 30% CSDG had almost no shift in peak, indicating that the crystal morphology was basically unchanged [35]. When the content was 20%, PLA/CSDG characteristic peaks shifted slightly to the right. However, when the CSDG content was 40–50%, the characteristic peak gradually shifted to the left. Evident self-aggregation may be the reason for this phenomenon. Similarly, in MPLA/CSDGG samples, the characteristic peak did not shift when the CSDG content was 10%. When the CSDG content was 20–30%, the characteristic peak gradually shifted to the right [36]. When CSDG promoted the completion of crystallization, and as the addition of CSDG was continued, the characteristic peak of MPLA shifted to the left. According to the Bragg equation: 2dsinθ = nλ, θ increases when nλ does not change, and the value of d decreases, which reduces the interplanar spacing d in a composite material, which may increase the crystallinity and increase the tensile strength. When CSDG was continually added, most CSDG particles occupied the PLA crystal array, so that θ decreased and d increased, resulting in decreased crystallinity. In other words, too many crystal nuclei may hinder the growth of crystals, which may lead to a decline in the degree of crystallization.

### 3.4. Morphological Images

**Figure 4:**
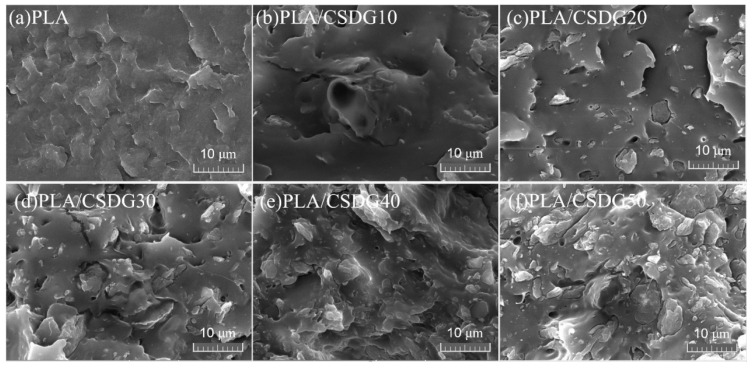
SEM images of PLA and PLA/CSDG composites: (**a**) PLA; (**b**) PLA/CSDG10; (**c**) PLA/CSDG20; (**d**) PLA/CSDG30; (**e**) PLA/CSDG40; (**f**) PLA/CSDG50.

Generally, when polymers deform under stress, the presence of fillers would produce concentration effects and cause micro-cracks around the polymer. The contact area of fillers with the polymer is large, and when the filler amount is small, micro-cracks would be generated. However, when the filler amount becomes too large, such micro-cracks would turn into macro-cracks, which should lead to poor mechanical properties [37]. Therefore, the shape characteristics of filler, the dispersion in the polymer matrix, and the adhesion between the filler and the matrix all have an important impact on the mechanical properties of composite materials [38,39,40,41]. SEM images of the tensile section of PLA/CSDG composites are shown in Figure 4. The surface of pure PLA (Figure 4a) was dense and uniform. It is observed that the fracture surface of PLA/CSDG composites exhibited a rough appearance, and the irregular appearance of CSDG particles interspersed and distributed in the section could be clearly observed. At higher CSDG concentrations (30, 40, 50 wt %), it is observed that the aggregates gradually increased, and it can be established that the dispersibility was better when the filler concentration was lower, and the interfacial effect was relatively good. This means that when the amount of CSDG was higher, poor interfacial adhesion would lead to uneven internal structure of the obtained PLA/CSDG composites, which may lead to deterioration of the mechanical properties [42].

**Figure 5:**
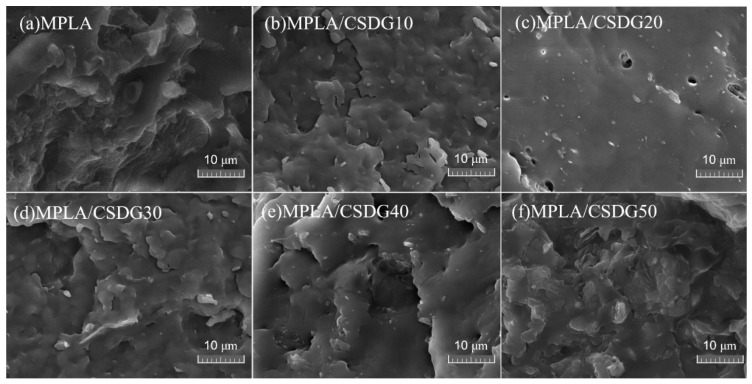
SEM images of MPLA and MPLA/CSDG composites: (**a**) MPLA; (**b**) MPLA/CSDG10; (**c**) MPLA/CSDG20; (**d**) MPLA/CSDG30; (**e**) MPLA/CSDG40; (**f**) MPLA/CSDG50.

Figure 5 illustrates the tensile section morphology of MPLA/CSDG samples with different amounts of CSDG. Although we can see that there are CSDG particles on the sample surface, MPLA/CSDG showed relatively flat morphology (relative to the rough surface of PLA/CSDG samples). Only a small amount of CSDG aggregates appeared in the matrix, and the distribution was relatively uniform. Nearly no gaps existed between the filler and the matrix, except for the sample with 20 wt % CSDG (Figure 5c). A small number of pores are visible, which may cause the elongation at break to be lower than that of the other samples. The relatively compact structure of MPLA/CSDG samples indicated that a dense structure was formed between MPLA and CSDG. In addition, when the CSDG content was 40 wt % (Figure 5e) and 50 wt % (Figure 5f), significant agglomeration occurred, which led to a decrease in the compatibility between CSDG and the MPLA matrix [43].

### 3.5. Thermal Stability Analysis

**Figure 6:**
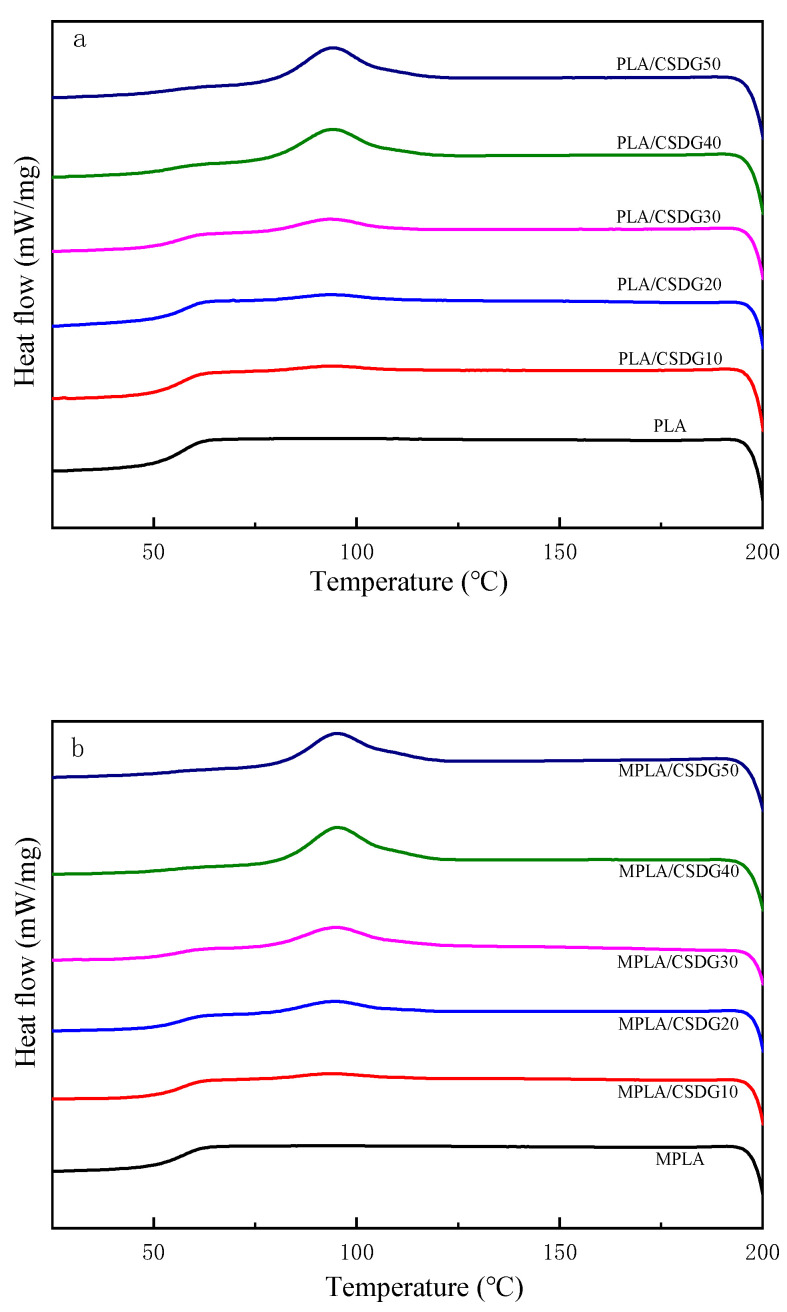
Cooling curves: (**a**) PLA/CSDG; (**b**) MPLA/CSDG.

**Figure 7:**
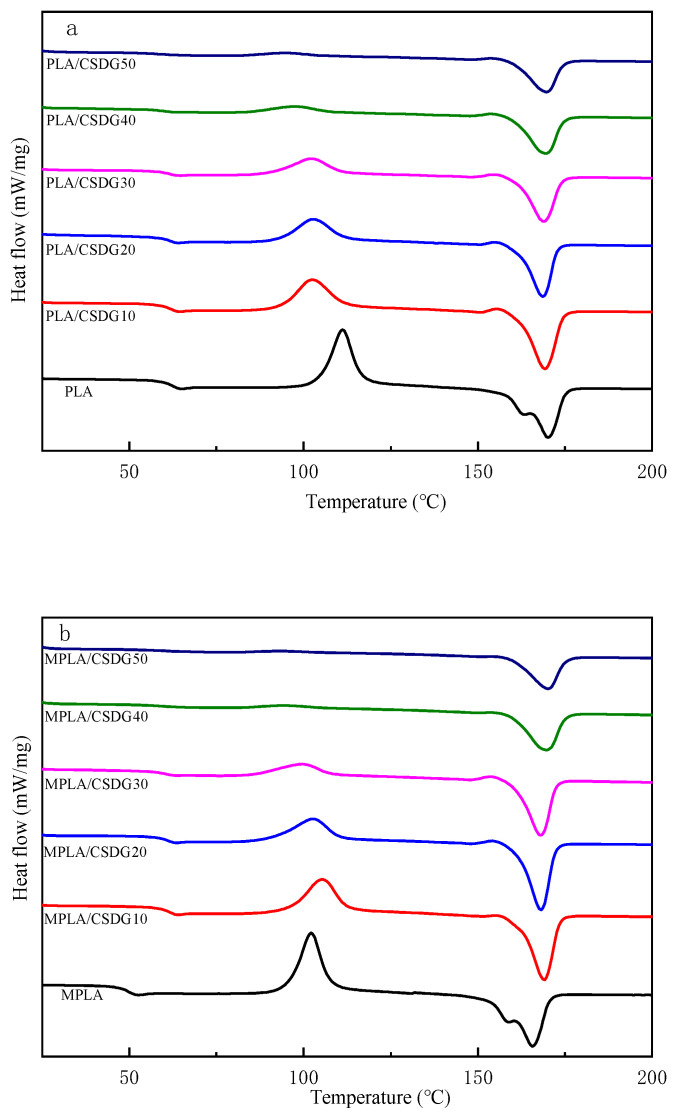
Second heating curves: (**a**) PLA/CSDG; (**b**) MPLA/CSDG.

DSC test results are shown in Figure 6, as well as in Table 3. DSC was conducted to determine the glass transition temperature (T_g_), melting temperature, recrystallization temperature (T_c_), enthalpy of fusion, enthalpy of crystallization, and degree of crystallinity of composite materials. From the cooling curves in Figure 6, PLA and MPLA indicated no crystallization peaks. This is because PLA had very slow crystallization, and the cooling rate of 10 °C/min was too fast, making it too late for PLA to crystallize [44]. With the addition of CSDG, both PLA and MPLA indicated crystallization peaks, which may be attributed to CSDG acting as a nucleating agent for polymers and accelerating the crystallization rate of PLA. From the first heating curves, because the samples were in the initial thermal history, the shape of the curves was irregular, so the sample second heating curves (Figure 7) were considered. In Figure 7, when the CSDG content gradually increased from 0 to 50%, T_g_ decreased from 68.7 to 58.6 °C, reaching the lowest temperature. The reason for the decrease in T_g_ may be the presence of many polar groups in CSDG itself. When the number of polar groups in the composite chain exceeded a certain value, the electrostatic repulsion between them exceeded the attractive force, which led to an increase in the distance between molecular chains and a decrease in T_g_. At the same time, the overall melting temperature of PLA/CSDG composites during the second heating was slightly lower than that of pure PLA, indicating that the rigidity of the blend was affected to a lesser extent, and the melting temperature was reduced from 170.1 to 168.2 °C (CSDG content increased from 0 to 20%), and it then began to rise (CSDG content, 20–50%). From the analysis, it is believed that when the CSDG content was too much (30%), so that it was not easy for CSDG to disperse in PLA, CSDG produced some subtle aggregation effects, making the crystallization less complete [45,46]. T_c_ decreased more significantly by 8–17 °C, indicating that CSDG exerted a nucleation effect on PLA [47]. The data for MPLA/CSDG samples showed that T_c_ and T_g_ were significantly lower than those for PLA/CSDG. When CSDG was added to PLA, due to the interaction between the hydroxyl groups in the PLA matrix and the filler, the degree of restriction in the movement of molecular chains was increased, hindering the movement of the polymer chains [48]. The existence of more functional groups in MPLA improved the molecular bonding between CSDG and MPLA as well as the uniform dispersion of CSDG in the MPLA matrix. The interaction between the MPLA matrix and the filler improved, free volume in the polymer increased, the degree of hindrance in the movement of molecular chains decreased, and the average chain length between cross-linking points became large, so T_g_ decreased.

### 3.6. Thermogravimetric Analysis

**Figure 8:**
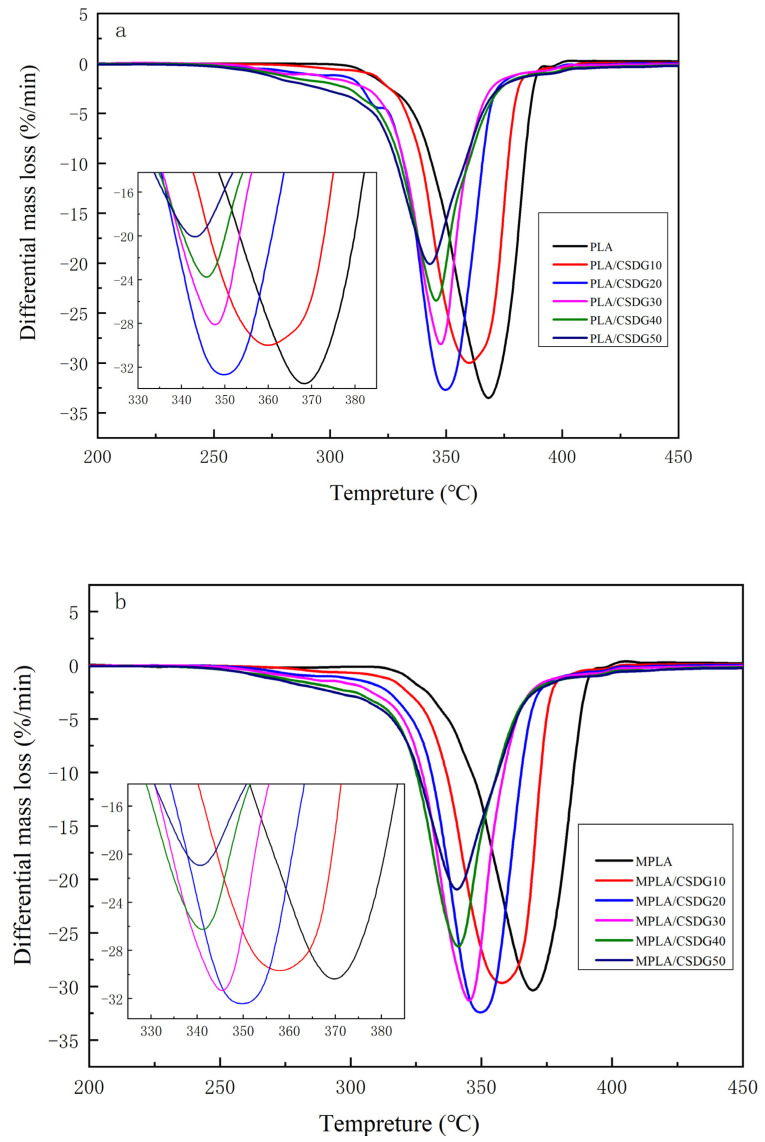
Differential thermogravimetric curves: (**a**) PLA/CSDG; (**b**) MPLA/CSDG.

DTG results for PLA/CSDG and MPLA/CSDG composites are shown in Figure 8. Table 4 lists the thermal degradation data. The thermal stability of composite materials was examined through TGA. PLA/CSDG and MPLA/CSDG composites both exhibited three stages of mass loss. First, mass loss started at around 200 °C due to the volatilization of water. The second step involved the loss of PLA at around 350 °C. The mass loss at about 450 °C in the third step was due to the decomposition of composite materials and modifiers. It can be deduced that the addition of CSDG fibers significantly reduced the initial decomposition temperature of the composite materials. A previous researcher analyzed the thermal stability of PLA/ramie composites and found that the addition of ramie fibers reduced the initial decomposition temperature of the composites [49], which is consistent with the finding from this present experiment. The initial degradation temperature of PLA/CSDG decreased with increase in the CSDG content. The temperature at maximum mass loss of PLA/CSDG decreased slightly compared with that of pure PLA. For MPLA/CSDG composites, the temperature at maximum mass loss decreased more significantly, and the initial degradation temperature also decreased substantially. This indicates that unmodified composites maintained better thermal stability, but the thermal stability of both modified and unmodified composites was lower compared with that of pure PLA. The thermal results agree with the formation of aggregates revealed by SEM images. Similar observations were detected for biopolymer matrices filled with coffee grounds [50] and inorganic clay nanoparticles [51]. In general, the clustering of fillers generates a reduction of the polymer thermal stability.

### 3.7. Analysis of Oxygen Barrier Performance

**Figure 9:**
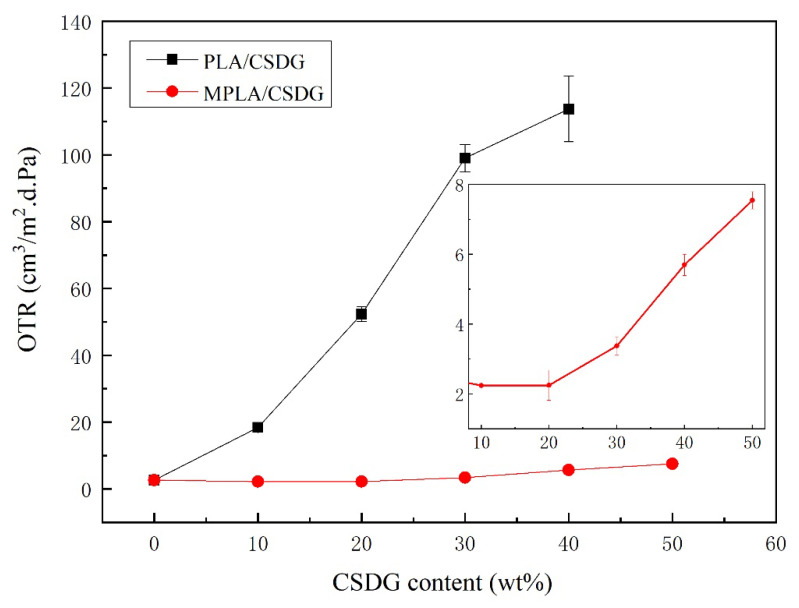
Oxygen barrier properties of PLA/CSDG and MPLA/CSDG composites.

Figure 9 describes the effect of different amounts of CSDG on the oxygen permeability in PLA/CSDG and MPLA/CSDG composites. The oxygen permeability in pure PLA was 2.626 cm^3^/m^2^·d·Pa. With the increase in the CSDG content, the trend in oxygen permeability increased. When the CSDG content was 40%, the oxygen permeability in PLA/CSDG increased to 113.725 cm^3^/m^2^·d·Pa. In particular, when the CSDG content was 50%, at which excessive self-aggregation of CSDG occurred and CSDG and PLA had poor compatibility with each other, the composite material exhibited too many defects and low mechanical properties. Therefore, we infer that due to the presence of a hydrophilic substance, more voids and micro-cracks were formed, resulting in the decreased oxygen barrier performance of PLA/CSDG. However, the oxygen barrier properties of MPLA/CSDG composites improved. When the CSDG content was 10%, the oxygen permeability in MPLA/CSDG dropped to 2.241 cm^3^/m^2^·d·Pa, which is slightly lower than that in pure PLA film. However, when the CSDG content was greater than 20%, the oxygen permeability started to increase. At the maximum CSDG content of 50%, the oxygen permeability in MPLA/CSDG was 7.551 cm^3^/m^2^·d·Pa, which was lower than that in PLA/CSDG (see sub-Figure 9). It is clear that MPLA had a significant effect on enhancing the oxygen barrier performance. Due to the acid anhydride group in MPLA, the composite material was able to form a relatively tight network structure and connections, leading to a significant decrease in oxygen permeability and a significant improvement in oxygen barrier performance.

### 3.8. Analysis of Water Vapor Barrier Properties

**Figure 10:**
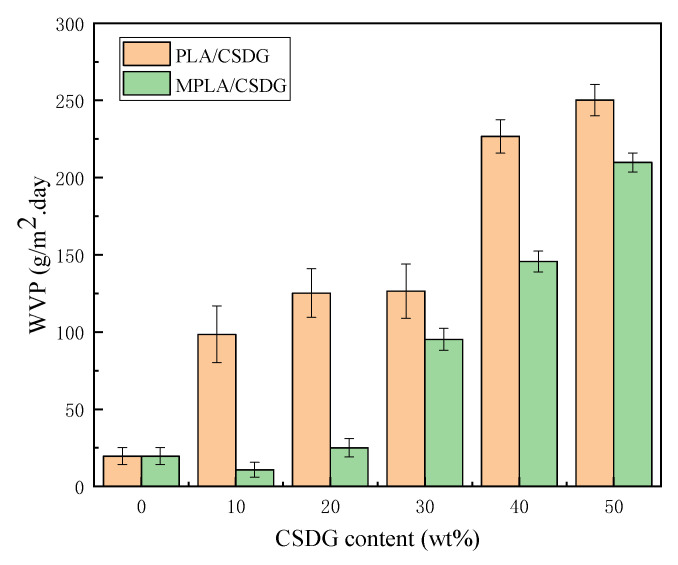
Water vapor barrier properties of PLA/CSDG and MPLA/CSDG composites.

**Figure 11:**
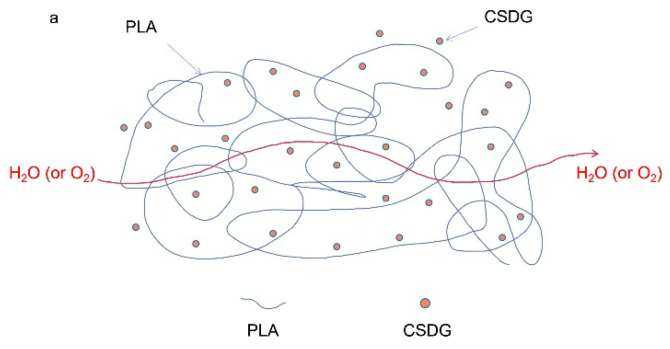

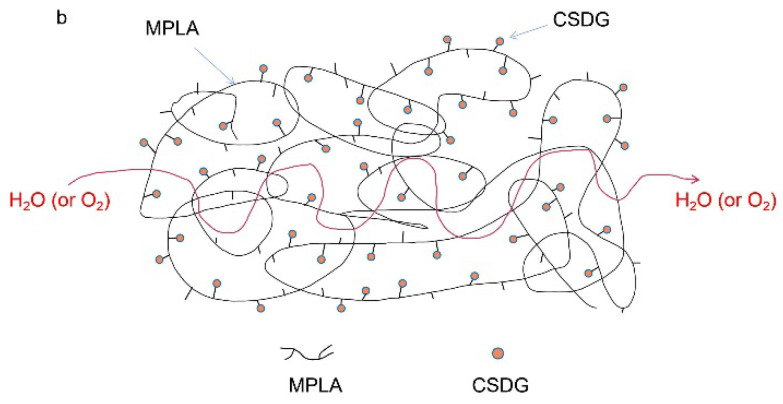
Schematic of molecular permeation path for water vapor or oxygen: (**a**) PLA/CSDG; (**b**) MPLA/CSDG.

Figure 10 plots test results about the influence of different amounts of CSDG on the water vapor permeability in PLA/CSDG and MPLA/CSDG composites. Due to the presence of a large amount of hydrophilic –OH in the molecular chain of PLA, the water-blocking performance of pure PLA film was poor. From the test results, the water vapor permeability in pure PLA was 19.64 g/m^2^/d. When CSDG was added, the water vapor permeability in PLA/CSDG began to increase. At 50% CSDG, the water vapor permeability in the composite film increased to 250.25 g/m^2^/d. Therefore, we infer that due to the CSDG hydrophilicity, as indicated from the analysis of electron microscope, excessive CSDG easily led to its agglomeration, and a large number of hydrophilic groups were exposed, thereby reducing the water vapor barrier properties of the composite material [52]. For the case of MPLA, especially when the CSDG content was 10%, the water vapor permeability in MPLA/CSDG dropped to 10.81 g/m^2^/d. When the added CSDG was 20%, the water vapor permeability in the MPLA/CSDG composite was higher than that in pure PLA film. When CSDG was 50%, the water vapor permeability reached 25.03 g/m^2^/d. These findings are consistent with the reported barrier properties of PLA-based composites [1]. However, this result for MPLA/CSDG is better than that for the PLA/CSDG composite films. The results showed that the introduction of the acid anhydride group in MPLA improved the adhesion between CSDG and the polymer, significantly improving the water vapor barrier properties of the composite material. However, at the same time, because the CSDG content was too high, agglomeration occurred, resulting in voids in the composite matrix, making the structure loose. This resulted in a decrease in the composite film resistance to water vapor permeability [53,54]. Figure 11 clearly depicts changes in the permeation path of water vapor or oxygen in PLA/CSDG and MPLA/CSDG composites. These findings are consistent with the reported barrier properties of PLA films [55]. However, due to the poor compatibility and poor dispersion of CSDG in the PLA matrix, PLA/CSDG samples failed to form a dense structure and a good barrier effect against water vapor or oxygen. Figure 11b indicates that MPLA/CSDG composites had denser structure, so it was more difficult for water or oxygen to pass through.

### 3.9. Contact Angle Data

**Figure 12:**
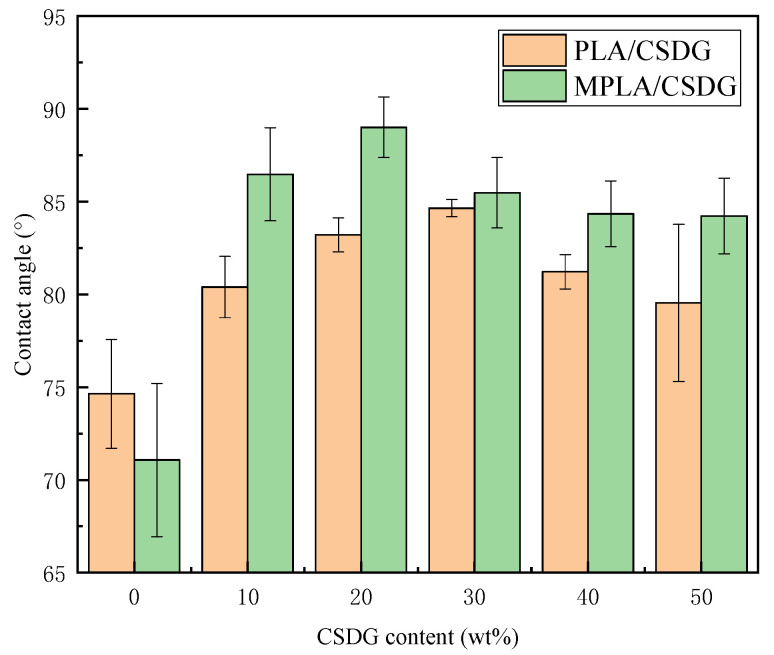
Contact angle of PLA/CSDG and MPLA/CSDG composites.

From the data analysis for PLA/CSDG and MPLA/CSDG composites in Figure 12, the contact angle of composites increased significantly. The overall contact angle of MPLA/CSDG was higher than that of unmodified composites. This can be explained by the reaction between the acid anhydride group in MPLA and –OH in CSDG to form a relatively tight network structure, making the material internal connections closer. The binding force at the interface between CSDG and PLA was enhanced, so that the hydrophobicity was also enhanced. With increase in the CSDG content, PLA/CSDG and MPLA/CSDG samples reached the highest contact angle when CSDG was 30% and 20%, respectively. At higher CSDG content, the contact angle began to decrease. The reason is the occurrence of CSDG agglomeration in the PLA matrix, suggesting that a certain maximum content of CSDG would cause the hydrophobicity of PLA to increase. PLA gave an initial contact angle of 73.5 ± 2.5°, while PLA/CSDG and MPLA/CSDG samples both showed higher contact angles. The maximum contact angle was close to 90° in the case of PLA/CSDG and 85° for MPLA/CSDG, indicating improvement in the hydrophobicity of PLA.

### 3.10. Water Absorption Analysis

**Figure 13:**
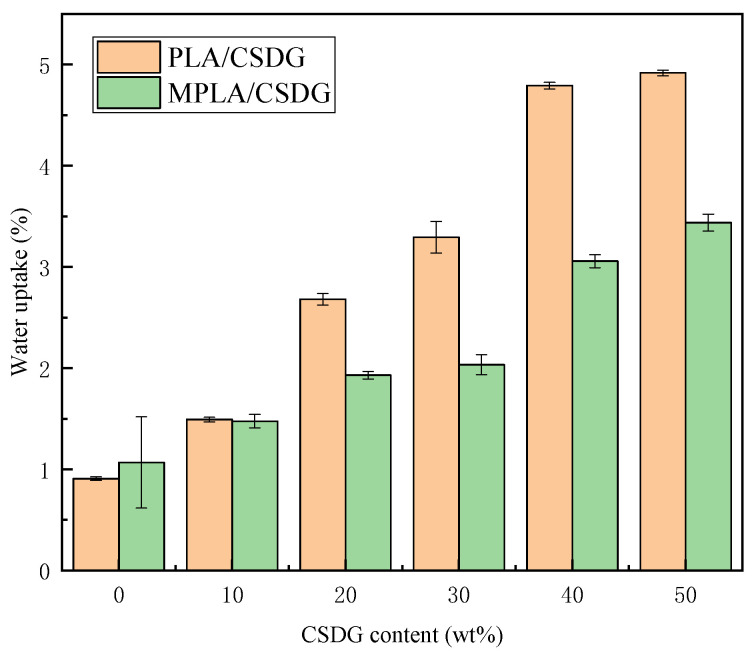
Water uptake for PLA/CSDG and MPLA/CSDG composites.

Test results on water absorption in PLA/CSDG and MPLA/CSDG composite films are indicated in Figure 13. It is clear that the rate of water absorption indicated a rising trend. This is because CSDG contained cellulose, hemicellulose, and lignin—all of them rich in hydroxyl groups—and therefore, materials with CSDG should have high water absorption. This same phenomenon is also reflected in the research by Wen et al. [56] on vinasse and polyethylene. The water absorption in MPLA/CSDG was lower than that in PLA/CSDG samples, and the increase in water absorption tended to be slow, indicating that MPLA/CSDG was more compact, so the water absorption was relatively poor. These results are consistent with the results of contact angle analysis. However, when the filler content was too high, CSDG would agglomerate, and the bond absorption capacity of hydroxyl groups on the surface of CSDG would increase. This may be the main reason for the increase in water absorption in samples with high CSDG content.

### 3.11. Biodegradation Rates

**Figure 14:**
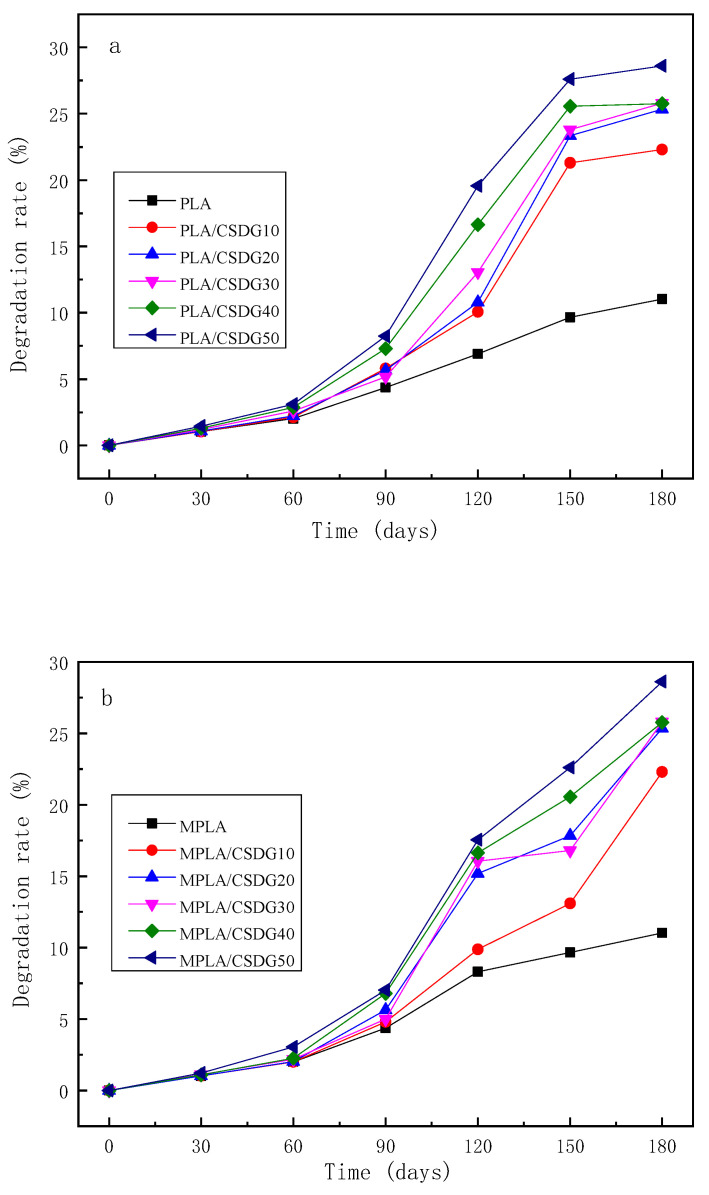
Degradability of (**a**) PLA/CSDG and (**b**) MPLA/CSDG composites.

Figure 14 presents a macroanalysis of PLA, MPLA, as well as PLA/CSDG and MPLA/CSDG composites during the process of degradation; changes in the biodegradation rates of different samples with time are shown. For all samples, the degradation could be roughly divided into two stages: initial stage (I) for the first 60 days, and final stage (II) after 60 days. In stage I, the sample biodegradation rate changed little with time. However, in stage II, changes in a short period of time were more observable, indicating that the sample degradation rate was greatly increased. In other words, the samples exhibited a “self-accelerating effect” during the biodegradation process [57]. The soil medium penetrated into the polymer matrix, causing the polymer molecular chains to relax, the chemical bonds to gradually decompose, the molecular weight to decrease, and the material to gradually degrade into oligomers. After 60 days of degradation, there would be more and more free hydroxyl (−OH) and carboxyl (−COOH) groups that accelerated their internal degradation, and further degradation would lead the oligomer to decompose into small molecules, resulting in increased degradation rate for the composite materials in later stages. It was observed that the degradation rate for MPLA/CSDG after 90 days was slightly lower than that for PLA/CSDG. On one hand, the MPLA composite exhibited relatively good internal compatibility. On the other hand, MPLA had reactive acid anhydride groups that would react with the hydroxyl groups on the surface of CSDG and with the carboxyl groups in the polymer matrix to increase the interfacial force between CSDG and MPLA, making the composite material relatively stable. Although the carboxyl group in the PLA matrix could also react with CSDG, the −COOH group was only at the end of the PLA molecular chain, which could not be compared with the large number of anhydride groups in the side chain of MPLA.

## 4. Conclusions

In this study, CSDG was incorporated as a reinforcing filler, and PLA/CSDG composites were prepared by melt blending. To improve the interfacial bonding force between CSDG and PLA, MPLA containing acid anhydride groups was adopted as well. Thus, MPLA/CSDG composites were also fabricated. For the two systems (PLA/CSDG and MPLA/CSDG), mechanical properties, hydrophobicity, gas barrier properties, biocompatibility, and thermodynamic properties were measured and comprehensively analyzed. The analysis revealed that the determining parameters were the dispersion of CSDG and the binding force between PLA and CSDG. Results demonstrated that with increasing CSDG content, the mechanical, barrier, hydrophobic, and thermal degradation properties all had corresponding changes that indicated different trends. The addition of MPLA improved the composite performance. Under the conditions considered in this work, the optimal composition of composites was 20–30% CSDG. In this composition range, MPLA/CSDG composites had good filler dispersion, enhanced hydrophobicity, improved stability, and high mechanical properties. The analysis from biodegradation experiments pointed out that as the degradation progressed, all the samples exhibited a “self-accelerating effect”. As the filler content increased, CSDG began to agglomerate, the brittleness of composites increased, and the barrier performance decreased. At the same time, MPLA/CSDG composites delivered stronger performance than PLA/CSDG composites. As CSDG is generally categorized as waste resources, recycling it not only reduces environmental issues but also expands the application of the natural biomass in environment-friendly functional materials.

## Figures and Tables

**Table 1:** Composition of PLA/CSDG and MPLA/CSDG composites.

Sample	PLA (%)	CSDG (%)	MPLA (%)
PLA/CSDG10	90	10	0
PLA/CSDG20	80	20	0
PLA/CSDG30	70	30	0
PLA/CSDG40	60	40	0
PLA/CSDG50	50	50	0
MPLA/CSDG10	0	10	90
MPLA/CSDG20	0	20	80
MPLA/CSDG30	0	30	70
MPLA/CSDG40	0	40	60
MPLA/CSDG50	0	50	50

**Table 2:** Data on mechanical properties of PLA, PLA/CSDG, MPLA, and MPLA/CSDG.

Sample	Tensile Strength (MPa)	Elongation at Break (%)
PLA	43.20 ± 1.9	5.08 ± 0.1
PLA/CSDG10	22.18 ± 2.9	2.43 ± 0.3
PLA/CSDG20	29.33 ± 2.8	2.80 ± 0.2
PLA/CSDG30	23.11 ± 1.9	3.05 ± 0.1
PLA/CSDG40	21.32 ± 2.2	2.60 ± 0.06
PLA/CSDG50	18.47 ± 2.1	2.35 ± 0.07
MPLA	37.08 ± 0.7	2.71 ± 0.3
MPLA/CSDG10	38.72 ± 1.9	2.75 ± 0.08
MPLA/CSDG20	41.13 ± 3.3	2.61 ± 0.4
MPLA/CSDG30	52.65 ± 2.0	6.02 ± 0.1
MPLA/CSDG40	30.30 ± 2.0	4.05 ± 0.5
MPLA/CSDG50	23.73 ± 1.8	2.72 ± 0.3

**Table 3:** Differential scanning calorimetry data for PLA, PLA/CSDG, MPLA, and MPLA/CSDG.

Sample	Glass Transition Temperature (°C)	Recrystallization Temperature (T_c_) (°C)	Enthalpy of Crystallization (J/g)	Melting Temperature (°C)	Melting Enthalpy (J/g)	Crystallinity (%)
PLA	61.7	111.2	29.79	170.1	31.84	37.0
PLA/CSDG10	61.2	102.5	21.67	169.3	32.67	38.74
PLA/CSDG20	61.1	102.8	16.77	168.2	26.12	34.84
PLA/CSDG30	61.1	102.2	11.82	169	24.6	37.51
PLA/CSDG40	59.9	97.9	7.692	169.5	23.1	40.93
PLA/CSDG50	58.6	94.8	3.198	169.7	19.4	41.43
MPLA	48.8	102.3	21.27	165.7	30.3	32.31
MPLA/SDG10	61.1	105.4	21.92	169	32.3	38.33
MPLA/SDG20	60.6	102.7	15.94	168.7	31.2	41.58
MPLA/SDG30	60.5	99.6	8.66	169.5	26.8	43.78
MPLA/SDG40	59.2	94.2	2.87	169.8	24.5	41.54
MPLA/CSDG50	59.5	93.6	1.51	170.1	20.3	41.22

**Table 4:** Differential thermogravimetric data for PLA, PLA/CSDG, MPLA, and MPLA/CSDG.

Sample	Initial Degradation Temperature (°C)	Temperature at Maximum Mass Loss (°C)	Mass Loss Rate (%)
PLA	311.98 ± 1.7	368.38 ± 0.9	67.33 ± 2.2
PLA/CSDG10	290.79 ± 1.5	360.21 ± 1.3	72.37 ± 3.5
PLA/CSDG20	269.85 ± 3.6	349.86 ± 1.1	60.70 ± 3.3
PLA/CSDG30	265.58 ± 0.9	347.89 ± 1.3	65.18 ± 4.1
PLA/CSDG40	257.58 ± 1.7	345.77 ± 0.8	56.52 ± 2.0
PLA/CSDG50	255.88 ± 1.9	342.99 ± 1.5	57.77 ± 2.9
MPLA	307.83 ± 0.4	368.70 ± 0.5	64.60 ± 1.6
MPLA/CSDG10	286.28 ± 1.4	357.61 ± 1.0	60.33 ± 3.0
MPLA/CSDG20	266.40 ± 1.0	349.25 ± 0.6	57.22 ± 2.1
MPLA/CSDG30	264.09 ± 0.7	345.19 ± 0.5	60.67 ± 2.8
MPLA/CSDG40	254.96 ± 1.8	341.13 ± 0.9	57.30 ± 2.5
MPLA/CSDG50	249.77 ± 0.9	340.52 ± 1.4	55.32 ± 2.9

## Data Availability

The data presented in this study are available on request from the corresponding author.
